# Tetra­kis(2,2′-bipyrid­yl)dichlorido­di-μ_3_-hydroxido-di-μ_2_-hydroxido-tetra­copper(II) dinitrate hexa­hydrate

**DOI:** 10.1107/S160053680804381X

**Published:** 2009-01-08

**Authors:** Ying Fan, Yong-Tao Cui, Hui-Fen Qian, Jian-Lan Liu, Wei Huang

**Affiliations:** a College of Sciences, Nanjing University of Technology, Nanjing, 210009, People’s Republic of China; b State Key Laboratory of Coordination Chemistry, Nanjing National Laboratory of Microstructures, School of Chemistry and Chemical Engineering, Nanjing University, Nanjing, 210093, People’s Republic of China

## Abstract

The tetra­nuclear copper(II) title complex, [Cu_4_Cl_2_(OH)_4_(C_10_H_8_N_2_)_4_](NO_3_)_2_·6H_2_O, has a crystallographically imposed centre of symmetry. The metal atoms display a distorted tetragonal-pyramidal coordination geometry, and are linked by two *μ*_2_- and two *μ_3_*-hydroxo groups, assuming a chair-like conformation for the Cu_4_O_2_ core. In the crystal, the complex mol­ecules are linked into a three-dimensional network by inter­molecular O—H⋯O, O—H⋯Cl, C—H⋯O and C—H⋯Cl hydrogen bonds and π–π stacking inter­actions with centroid–centroid separations of 3.724 (2) and 3.767 (3) Å.

## Related literature

For the structures of related complexes, see: Albada *et al.* (2002 ▶); Chandrasekhar *et al.* (2000 ▶); Lu *et al.* (2007 ▶); Sletten *et al.* (1990 ▶); Zheng & Lin (2002 ▶).
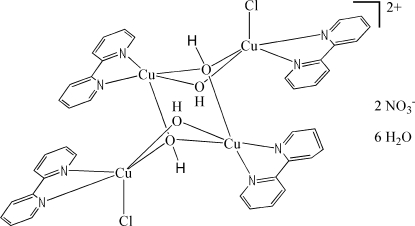


## Experimental

### 

#### Crystal data

[Cu_4_Cl_2_(OH)_4_(C_10_H_8_N_2_)_4_](NO_3_)_2_·6H_2_O
*M* *_r_* = 1249.94
Triclinic, 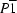

*a* = 9.389 (3) Å
*b* = 10.622 (3) Å
*c* = 12.950 (4) Å
α = 86.909 (4)°
β = 77.263 (3)°
γ = 72.512 (4)°
*V* = 1201.4 (6) Å^3^
*Z* = 1
Mo *K*α radiation
μ = 1.94 mm^−1^
*T* = 291 (2) K
0.16 × 0.12 × 0.10 mm

#### Data collection

Bruker SMART CCD area-detector diffractometer
Absorption correction: multi-scan (*SADABS*; Bruker, 2000 ▶) *T*_min_ = 0.747, *T*_max_ = 0.830
6088 measured reflections
4181 independent reflections
3156 reflections with *I* > 2σ(*I*)
*R*_int_ = 0.025

#### Refinement

*R*[*F* ^2^ > 2σ(*F* ^2^)] = 0.047
*wR*(*F* ^2^) = 0.153
*S* = 1.08
4181 reflections
319 parameters
H-atom parameters constrained
Δρ_max_ = 0.94 e Å^−3^
Δρ_min_ = −1.04 e Å^−3^

### 

Data collection: *SMART* (Bruker, 2000 ▶); cell refinement: *SAINT* (Bruker, 2000 ▶); data reduction: *SAINT*; program(s) used to solve structure: *SHELXTL* (Sheldrick, 2008 ▶); program(s) used to refine structure: *SHELXTL*; molecular graphics: *SHELXTL*; software used to prepare material for publication: *SHELXTL*.

## Supplementary Material

Crystal structure: contains datablocks global, I. DOI: 10.1107/S160053680804381X/rz2280sup1.cif

Structure factors: contains datablocks I. DOI: 10.1107/S160053680804381X/rz2280Isup2.hkl

Additional supplementary materials:  crystallographic information; 3D view; checkCIF report

## Figures and Tables

**Table 1:** Selected bond lengths (Å)

Cu1—O2	1.927 (3)
Cu1—O1	1.980 (3)
Cu1—N1	2.016 (4)
Cu1—N2	2.029 (4)
Cu1—Cl1	2.5942 (17)
Cu2—O2	1.924 (3)
Cu2—O1	1.959 (3)
Cu2—N4	1.989 (4)
Cu2—N3	2.012 (4)
Cu2—O1^i^	2.323 (3)

Symmetry code: (i) 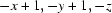.

**Table 2:** Hydrogen-bond geometry (Å, °)

*D*—H⋯*A*	*D*—H	H⋯*A*	*D*⋯*A*	*D*—H⋯*A*
O1—H1*A*⋯O4	0.85	2.02	2.835 (7)	160
O2—H2*A*⋯O6	0.85	2.28	2.874 (6)	127
O7—H7*A*⋯O8^ii^	0.85	2.17	2.714 (9)	121
O8—H8*A*⋯Cl1	0.85	2.39	3.187 (7)	157
C2—H2⋯Cl1^iii^	0.93	2.82	3.692 (5)	156
C5—H5⋯O4	0.93	2.55	3.394 (7)	152
C10—H10⋯O6	0.93	2.46	3.318 (7)	154
C12—H12⋯Cl1^iv^	0.93	2.78	3.679 (5)	162
C15—H15⋯O4	0.93	2.58	3.185 (8)	123

Symmetry codes: (ii) 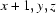; (iii) 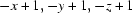; (iv) 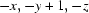.

**Fig. 1.**
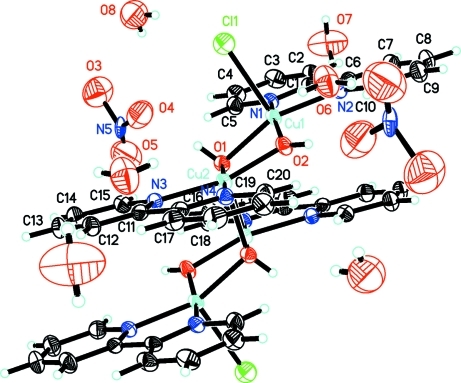
The structure of the title compound with the atom-numbering scheme. Displacement ellipsoids are drawn at the 30% probability level. Unlabeled atoms are related to the labeled atoms by (1-x, 1-y, -z).

**Fig. 2.**
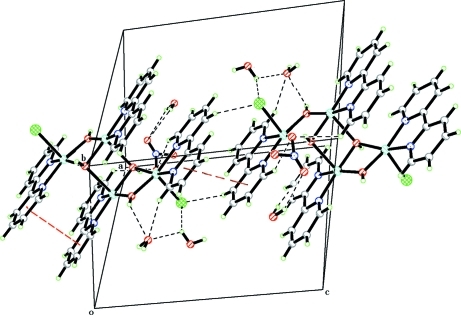
Perspective view of the crystal packing the title compound showing the the hydrogen bonds and π–π stacking interactions as dashed lines.

## supplementary crystallographic information

### Comment

Recently, some tetranuclear hydroxo-bridged copper(II) complexes with cubane and the chair-like structure have been reported (Zheng & Lin, 2002; Sletten *et al.*, 1990; Albada *et al.*, 2002; Lu *et al.*, 2007; Chandrasekhar *et al.*, 2000). In this paper, the crystal structure of a new copper(II) complex exhibiting a chair-like tetranuclear motif is presented.

The atom-numbering scheme of the title compound is shown in Fig. 1, while selected bond distances are given in Table 1. The title complex has a crystallographically imposed centre of symmetry, and consists of a chair-like [Cu_4_(bpy)_4_(*µ*_2_-OH)_2_(*µ*_3_-OH)_2_Cl_2_]^2+^ dication (bpy = 2,2'-bipyridine), two nitrate anions, and six lattice water molecules. The coordination geometry around each copper(II) ion can be described as a five-coordinate distorted pyramid. The basal sites are occupied by two N atoms from a bpy ligand and two O atoms from two *µ*_2_-bridging hydroxo groups, with mean Cu–N and Cu–O bond distances of 2.011 (4) 1.948 (3) Å, respectively; the apical position is occupied by a chloride anion for atom Cu1 (Cu1–Cl1 = 2.594 (2) Å) and a *µ*_3_-bridged OH anion for Cu2 (Cu2–O1^i^ = 2.323 (3) Å; symmetry code: (i) = 1-x, 1-y, -z).

In the crystal packing, the complex molecules are linked into a three-dimensional network by intra- and intermolecular O—H···O, O—H···Cl, C—H···O and C—H···Cl hydrogen bonding interactions involving the solvent water molecules, the hydroxo groups and the chloride and nitrate anions (Table 2). The structure is further stabilized by π–π stacking interactions between adjacent bpy molecules with centroid-to-centroid separations of 3.724 (2) and 3.767 (3) Å (Fig. 2).

### Experimental

The title compound was obtained as a by-product from the reaction between [Cu(bpy)](NO_3_)_2_ (0.398 g, 1 mmol) and D-(+)-1,2,2-trimethylcyclopentane-1,3-diamine dihydrogenchloride salt (0.284 g, 2 mmol) in the presence of NaOH (0.080 g, 2 mmol). Yield: 35 % based on the copper(II) amount. Single crystals suitable for X-ray diffraction were grown from a mixture of methanol/water (1:1 v/v) by slow evaporation in air at room temperature. Elemental Analysis: Calcd. for C_40_H_48_Cl_2_Cu_4_N_10_O_16_: C, 38.44; H, 3.87; N, 11.21 %; found: C,38.66; H,3.67; N, 11.03 %. Main FT-IR absorptions (KBr pellets, cm^-1^): 3427 (vs), 2372 (m), 2341 (m), 1634 (s), 1383 (m), 1080 (s), 991 (m), 773 (m), and 549 (m).

### Refinement

All H atoms were placed in geometrically idealized positions and refined as riding, with C—H = 0.93 Å, O—H = 0.85 Å, and with *U*_iso_(H) = 1.2*U*_eq_(C) or 1.5*U*_eq_(O).

### Crystal data

**Table:**

[Cu_4_Cl_2_(OH)_4_(C_10_H_8_N_2_)_4_](NO_3_)_2_·6H_2_O	*Z* = 1
*M**_r_* = 1249.94	*F*(000) = 636
Triclinic, *P*1	*D*_x_ = 1.728 Mg m^−^^3^
Hall symbol: -P 1	Mo *K*α radiation, λ = 0.71073 Å
*a* = 9.389 (3) Å	Cell parameters from 2700 reflections
*b* = 10.622 (3) Å	θ = 2.3–27.2°
*c* = 12.950 (4) Å	µ = 1.94 mm^−^^1^
α = 86.909 (4)°	*T* = 291 K
β = 77.263 (3)°	Block, blue
γ = 72.512 (4)°	0.16 × 0.12 × 0.10 mm
*V* = 1201.4 (6) Å^3^	

### Data collection

**Table:**

Bruker SMART CCD area-detector diffractometer	4181 independent reflections
Radiation source: fine-focus sealed tube	3156 reflections with *I* > 2σ(*I*)
graphite	*R*_int_ = 0.025
φ and ω scans	θ_max_ = 25.0°, θ_min_ = 2.3°
Absorption correction: multi-scan (*SADABS*; Bruker, 2000)	*h* = −10→11
*T*_min_ = 0.747, *T*_max_ = 0.830	*k* = −12→12
6088 measured reflections	*l* = −15→13

### Refinement

**Table:**

Refinement on *F*^2^	Primary atom site location: structure-invariant direct methods
Least-squares matrix: full	Secondary atom site location: difference Fourier map
*R*[*F*^2^ > 2σ(*F*^2^)] = 0.047	Hydrogen site location: inferred from neighbouring sites
*wR*(*F*^2^) = 0.153	H-atom parameters constrained
*S* = 1.08	*w* = 1/[σ^2^(*F*_o_^2^) + (0.0958*P*)^2^] where *P* = (*F*_o_^2^ + 2*F*_c_^2^)/3
4181 reflections	(Δ/σ)_max_ < 0.001
319 parameters	Δρ_max_ = 0.94 e Å^−^^3^
0 restraints	Δρ_min_ = −1.04 e Å^−^^3^

### Special details

**Table:**

Experimental. The structure was solved by direct methods (Bruker, 2000) and successive difference Fourier syntheses.
Geometry. All e.s.d.'s (except the e.s.d. in the dihedral angle between two l.s. planes) are estimated using the full covariance matrix. The cell e.s.d.'s are taken into account individually in the estimation of e.s.d.'s in distances, angles and torsion angles; correlations between e.s.d.'s in cell parameters are only used when they are defined by crystal symmetry. An approximate (isotropic) treatment of cell e.s.d.'s is used for estimating e.s.d.'s involving l.s. planes.
Refinement. Refinement of *F*^2^ against ALL reflections. The weighted *R*-factor *wR* and goodness of fit *S* are based on *F*^2^, conventional *R*-factors *R* are based on *F*, with *F* set to zero for negative *F*^2^. The threshold expression of *F*^2^ > σ(*F*^2^) is used only for calculating *R*-factors(gt) *etc*. and is not relevant to the choice of reflections for refinement. *R*-factors based on *F*^2^ are statistically about twice as large as those based on *F*, and *R*- factors based on ALL data will be even larger.

### Fractional atomic coordinates and isotropic or equivalent isotropic displacement parameters (Å^2^)

**Table:**

	*x*	*y*	*z*	*U*_iso_*/*U*_eq_	
Cu1	0.46249 (6)	0.44695 (6)	0.22215 (4)	0.03567 (19)	
Cu2	0.36457 (6)	0.43378 (5)	0.02528 (4)	0.03328 (18)	
C1	0.6144 (5)	0.5218 (5)	0.3666 (3)	0.0348 (10)	
C2	0.6642 (6)	0.5990 (6)	0.4245 (4)	0.0490 (13)	
H2	0.7294	0.5603	0.4696	0.059*	
C3	0.6160 (7)	0.7337 (6)	0.4146 (4)	0.0528 (14)	
H3	0.6491	0.7872	0.4526	0.063*	
C4	0.5183 (6)	0.7893 (6)	0.3479 (4)	0.0509 (13)	
H4	0.4827	0.8804	0.3414	0.061*	
C5	0.4755 (6)	0.7075 (5)	0.2917 (4)	0.0453 (12)	
H5	0.4116	0.7447	0.2455	0.054*	
C6	0.6549 (5)	0.3764 (5)	0.3726 (3)	0.0366 (11)	
C7	0.7526 (6)	0.3022 (6)	0.4334 (4)	0.0507 (13)	
H7	0.7974	0.3424	0.4740	0.061*	
C8	0.7825 (7)	0.1664 (6)	0.4324 (5)	0.0602 (15)	
H8	0.8485	0.1143	0.4725	0.072*	
C9	0.7158 (7)	0.1094 (6)	0.3732 (4)	0.0547 (14)	
H9	0.7348	0.0183	0.3724	0.066*	
C10	0.6195 (6)	0.1888 (5)	0.3144 (4)	0.0458 (12)	
H10	0.5734	0.1500	0.2737	0.055*	
C11	0.1473 (5)	0.5060 (5)	−0.1073 (4)	0.0372 (11)	
C12	0.0421 (6)	0.5816 (6)	−0.1627 (4)	0.0481 (13)	
H12	−0.0076	0.5418	−0.1997	0.058*	
C13	0.0117 (6)	0.7171 (6)	−0.1622 (4)	0.0537 (14)	
H13	−0.0596	0.7695	−0.1985	0.064*	
C14	0.0865 (6)	0.7741 (6)	−0.1082 (4)	0.0499 (13)	
H14	0.0670	0.8653	−0.1072	0.060*	
C15	0.1916 (6)	0.6937 (5)	−0.0553 (4)	0.0432 (12)	
H15	0.2442	0.7321	−0.0198	0.052*	
C16	0.1902 (5)	0.3603 (5)	−0.1028 (3)	0.0350 (10)	
C17	0.1309 (6)	0.2829 (6)	−0.1531 (4)	0.0474 (13)	
H17	0.0568	0.3218	−0.1918	0.057*	
C18	0.1819 (6)	0.1486 (6)	−0.1455 (4)	0.0518 (14)	
H18	0.1422	0.0953	−0.1785	0.062*	
C19	0.2941 (6)	0.0924 (5)	−0.0878 (4)	0.0507 (13)	
H19	0.3314	0.0012	−0.0824	0.061*	
C20	0.3483 (6)	0.1740 (5)	−0.0393 (4)	0.0448 (12)	
H20	0.4228	0.1365	−0.0007	0.054*	
Cl1	0.18510 (17)	0.49867 (15)	0.33336 (11)	0.059	
N1	0.5207 (4)	0.5767 (4)	0.3000 (3)	0.0360 (9)	
N2	0.5897 (4)	0.3206 (4)	0.3135 (3)	0.0372 (9)	
N3	0.2209 (4)	0.5615 (4)	−0.0532 (3)	0.0351 (9)	
N4	0.2985 (4)	0.3057 (4)	−0.0451 (3)	0.0362 (9)	
N5	0.1809 (5)	0.8933 (4)	0.1658 (4)	0.0347 (10)	
O1	0.4182 (4)	0.5621 (3)	0.1006 (2)	0.0344 (7)	
H1A	0.3305	0.6140	0.1271	0.052*	
O2	0.4539 (4)	0.3165 (3)	0.1278 (2)	0.0408 (8)	
H2A	0.4821	0.2327	0.1302	0.061*	
O3	0.0821 (8)	0.9740 (6)	0.2427 (6)	0.130 (2)	
O4	0.1649 (6)	0.7823 (6)	0.1809 (5)	0.1076 (18)	
O5	0.2665 (11)	0.9185 (7)	0.1204 (6)	0.140 (3)	
O6	0.3529 (6)	0.1021 (5)	0.2300 (4)	0.0985 (17)	
H6A	0.3847	0.0186	0.2223	0.148*	
H6B	0.2923	0.1204	0.1876	0.148*	
O7	0.8224 (8)	0.7897 (8)	0.5938 (5)	0.156 (3)	
H7A	0.8445	0.7449	0.5370	0.235*	
H7B	0.7521	0.7659	0.6357	0.235*	
O8	−0.0164 (7)	0.7957 (7)	0.3929 (5)	0.124 (2)	
H8A	0.0125	0.7120	0.3871	0.185*	
H8B	0.0606	0.8244	0.3741	0.185*	

### Atomic displacement parameters (Å^2^)

**Table:**

	*U*^11^	*U*^22^	*U*^33^	*U*^12^	*U*^13^	*U*^23^
Cu1	0.0413 (4)	0.0418 (4)	0.0281 (3)	−0.0113 (3)	−0.0174 (2)	0.0003 (2)
Cu2	0.0359 (3)	0.0396 (3)	0.0297 (3)	−0.0123 (3)	−0.0165 (2)	0.0008 (2)
C1	0.033 (2)	0.051 (3)	0.026 (2)	−0.017 (2)	−0.0108 (18)	0.001 (2)
C2	0.053 (3)	0.068 (4)	0.036 (3)	−0.023 (3)	−0.023 (2)	0.002 (2)
C3	0.066 (4)	0.063 (4)	0.044 (3)	−0.032 (3)	−0.022 (3)	−0.002 (3)
C4	0.060 (3)	0.049 (3)	0.048 (3)	−0.019 (3)	−0.015 (3)	0.000 (2)
C5	0.045 (3)	0.050 (3)	0.043 (3)	−0.009 (2)	−0.020 (2)	−0.002 (2)
C6	0.032 (2)	0.048 (3)	0.028 (2)	−0.006 (2)	−0.0087 (19)	0.000 (2)
C7	0.051 (3)	0.061 (4)	0.046 (3)	−0.014 (3)	−0.028 (3)	0.004 (3)
C8	0.054 (4)	0.067 (4)	0.056 (4)	−0.003 (3)	−0.030 (3)	0.014 (3)
C9	0.062 (4)	0.047 (3)	0.053 (3)	−0.007 (3)	−0.022 (3)	0.007 (3)
C10	0.051 (3)	0.044 (3)	0.040 (3)	−0.006 (2)	−0.015 (2)	−0.007 (2)
C11	0.028 (2)	0.051 (3)	0.031 (2)	−0.009 (2)	−0.0067 (19)	0.000 (2)
C12	0.036 (3)	0.071 (4)	0.041 (3)	−0.016 (3)	−0.019 (2)	0.005 (3)
C13	0.040 (3)	0.062 (4)	0.053 (3)	0.000 (3)	−0.020 (2)	0.014 (3)
C14	0.049 (3)	0.047 (3)	0.052 (3)	−0.008 (3)	−0.017 (2)	0.009 (2)
C15	0.046 (3)	0.045 (3)	0.036 (3)	−0.009 (2)	−0.011 (2)	0.002 (2)
C16	0.029 (2)	0.049 (3)	0.029 (2)	−0.015 (2)	−0.0062 (18)	0.000 (2)
C17	0.044 (3)	0.065 (4)	0.042 (3)	−0.023 (3)	−0.015 (2)	−0.006 (3)
C18	0.053 (3)	0.061 (4)	0.053 (3)	−0.028 (3)	−0.016 (3)	−0.010 (3)
C19	0.062 (4)	0.043 (3)	0.052 (3)	−0.019 (3)	−0.016 (3)	−0.003 (2)
C20	0.044 (3)	0.046 (3)	0.044 (3)	−0.008 (2)	−0.015 (2)	−0.001 (2)
Cl1	0.059	0.067	0.055	−0.021	−0.019	−0.008
N1	0.039 (2)	0.041 (2)	0.031 (2)	−0.0119 (18)	−0.0130 (16)	0.0004 (17)
N2	0.036 (2)	0.046 (2)	0.029 (2)	−0.0080 (18)	−0.0118 (16)	−0.0009 (17)
N3	0.030 (2)	0.045 (2)	0.032 (2)	−0.0100 (18)	−0.0110 (16)	−0.0012 (17)
N4	0.037 (2)	0.042 (2)	0.033 (2)	−0.0127 (18)	−0.0127 (17)	0.0019 (17)
N5	0.029 (2)	0.0156 (19)	0.066 (3)	−0.0057 (17)	−0.026 (2)	0.0040 (19)
O1	0.0386 (18)	0.0388 (18)	0.0292 (16)	−0.0108 (14)	−0.0145 (13)	−0.0016 (13)
O2	0.053 (2)	0.0387 (19)	0.0348 (18)	−0.0112 (16)	−0.0226 (15)	0.0014 (14)
O3	0.139 (6)	0.086 (4)	0.152 (6)	−0.031 (4)	−0.007 (5)	−0.010 (4)
O4	0.085 (4)	0.118 (5)	0.110 (5)	−0.015 (3)	−0.019 (3)	−0.019 (4)
O5	0.184 (8)	0.117 (6)	0.110 (6)	−0.033 (6)	−0.031 (5)	0.004 (4)
O6	0.088 (4)	0.093 (4)	0.132 (5)	−0.043 (3)	−0.046 (3)	0.039 (3)
O7	0.159 (6)	0.232 (9)	0.119 (5)	−0.129 (6)	−0.004 (5)	−0.040 (5)
O8	0.112 (5)	0.149 (6)	0.101 (5)	−0.018 (4)	−0.031 (4)	−0.008 (4)

### Geometric parameters (Å, °)

**Table:**

Cu1—O2	1.927 (3)	C11—C12	1.382 (7)
Cu1—O1	1.980 (3)	C11—C16	1.479 (7)
Cu1—N1	2.016 (4)	C12—C13	1.381 (8)
Cu1—N2	2.029 (4)	C12—H12	0.9300
Cu1—Cl1	2.5942 (17)	C13—C14	1.366 (8)
Cu2—O2	1.924 (3)	C13—H13	0.9300
Cu2—O1	1.959 (3)	C14—C15	1.379 (7)
Cu2—N4	1.989 (4)	C14—H14	0.9300
Cu2—N3	2.012 (4)	C15—N3	1.347 (6)
Cu2—O1^i^	2.323 (3)	C15—H15	0.9300
C1—N1	1.352 (5)	C16—N4	1.364 (6)
C1—C2	1.380 (6)	C16—C17	1.380 (6)
C1—C6	1.478 (7)	C17—C18	1.367 (8)
C2—C3	1.373 (8)	C17—H17	0.9300
C2—H2	0.9300	C18—C19	1.396 (7)
C3—C4	1.381 (7)	C18—H18	0.9300
C3—H3	0.9300	C19—C20	1.367 (7)
C4—C5	1.361 (7)	C19—H19	0.9300
C4—H4	0.9300	C20—N4	1.338 (6)
C5—N1	1.331 (6)	C20—H20	0.9300
C5—H5	0.9300	N5—O5	0.983 (8)
C6—N2	1.336 (6)	N5—O4	1.233 (7)
C6—C7	1.378 (7)	N5—O3	1.339 (7)
C7—C8	1.384 (8)	O1—Cu2^i^	2.323 (3)
C7—H7	0.9300	O1—H1A	0.8500
C8—C9	1.357 (8)	O2—H2A	0.8501
C8—H8	0.9300	O6—H6A	0.8501
C9—C10	1.373 (7)	O6—H6B	0.8498
C9—H9	0.9300	O7—H7A	0.8499
C10—N2	1.342 (6)	O7—H7B	0.8501
C10—H10	0.9300	O8—H8A	0.8499
C11—N3	1.349 (6)	O8—H8B	0.8500
			
O2—Cu1—O1	81.23 (13)	C13—C12—H12	120.5
O2—Cu1—N1	166.66 (15)	C11—C12—H12	120.5
O1—Cu1—N1	96.22 (14)	C14—C13—C12	119.9 (5)
O2—Cu1—N2	97.19 (15)	C14—C13—H13	120.1
O1—Cu1—N2	157.97 (15)	C12—C13—H13	120.1
N1—Cu1—N2	80.23 (15)	C13—C14—C15	118.6 (5)
O2—Cu1—Cl1	98.14 (11)	C13—C14—H14	120.7
O1—Cu1—Cl1	98.03 (10)	C15—C14—H14	120.7
N1—Cu1—Cl1	95.17 (12)	N3—C15—C14	122.5 (5)
N2—Cu1—Cl1	103.92 (11)	N3—C15—H15	118.8
O2—Cu2—O1	81.86 (13)	C14—C15—H15	118.8
O2—Cu2—N4	97.99 (15)	N4—C16—C17	121.4 (5)
O1—Cu2—N4	176.65 (14)	N4—C16—C11	114.1 (4)
O2—Cu2—N3	165.08 (15)	C17—C16—C11	124.5 (4)
O1—Cu2—N3	98.37 (14)	C18—C17—C16	119.4 (5)
N4—Cu2—N3	80.91 (15)	C18—C17—H17	120.3
O2—Cu2—O1^i^	100.99 (13)	C16—C17—H17	120.3
O1—Cu2—O1^i^	83.97 (12)	C17—C18—C19	119.3 (5)
N4—Cu2—O1^i^	99.33 (13)	C17—C18—H18	120.3
N3—Cu2—O1^i^	93.86 (13)	C19—C18—H18	120.3
N1—C1—C2	121.1 (5)	C20—C19—C18	118.6 (5)
N1—C1—C6	114.9 (4)	C20—C19—H19	120.7
C2—C1—C6	124.0 (4)	C18—C19—H19	120.7
C3—C2—C1	119.0 (5)	N4—C20—C19	122.8 (5)
C3—C2—H2	120.5	N4—C20—H20	118.6
C1—C2—H2	120.5	C19—C20—H20	118.6
C2—C3—C4	119.6 (5)	C5—N1—C1	118.9 (4)
C2—C3—H3	120.2	C5—N1—Cu1	126.2 (3)
C4—C3—H3	120.2	C1—N1—Cu1	114.9 (3)
C5—C4—C3	118.4 (5)	C6—N2—C10	118.9 (4)
C5—C4—H4	120.8	C6—N2—Cu1	115.1 (3)
C3—C4—H4	120.8	C10—N2—Cu1	125.9 (3)
N1—C5—C4	123.0 (5)	C15—N3—C11	118.5 (4)
N1—C5—H5	118.5	C15—N3—Cu2	126.6 (3)
C4—C5—H5	118.5	C11—N3—Cu2	114.9 (3)
N2—C6—C7	121.8 (5)	C20—N4—C16	118.4 (4)
N2—C6—C1	114.7 (4)	C20—N4—Cu2	126.2 (3)
C7—C6—C1	123.5 (4)	C16—N4—Cu2	115.4 (3)
C6—C7—C8	118.4 (5)	O5—N5—O4	128.5 (7)
C6—C7—H7	120.8	O5—N5—O3	121.4 (6)
C8—C7—H7	120.8	O4—N5—O3	108.1 (5)
C9—C8—C7	120.1 (5)	Cu2—O1—Cu1	95.59 (13)
C9—C8—H8	119.9	Cu2—O1—Cu2^i^	96.03 (12)
C7—C8—H8	119.9	Cu1—O1—Cu2^i^	113.66 (14)
C8—C9—C10	118.7 (5)	Cu2—O1—H1A	101.5
C8—C9—H9	120.7	Cu1—O1—H1A	101.5
C10—C9—H9	120.7	Cu2^i^—O1—H1A	138.7
N2—C10—C9	122.2 (5)	Cu2—O2—Cu1	98.51 (15)
N2—C10—H10	118.9	Cu2—O2—H2A	130.7
C9—C10—H10	118.9	Cu1—O2—H2A	130.8
N3—C11—C12	121.5 (5)	H6A—O6—H6B	99.3
N3—C11—C16	114.6 (4)	H7A—O7—H7B	106.7
C12—C11—C16	123.9 (4)	H8A—O8—H8B	109.5
C13—C12—C11	119.0 (5)		

Symmetry codes: (i) −*x*+1, −*y*+1, −*z*.

### Hydrogen-bond geometry (Å, °)

**Table:**

*D*—H···*A*	*D*—H	H···*A*	*D*···*A*	*D*—H···*A*
O1—H1A···O4	0.85	2.02	2.835 (7)	160
O2—H2A···O6	0.85	2.28	2.874 (6)	127
O7—H7A···O8^ii^	0.85	2.17	2.714 (9)	121
O8—H8A···Cl1	0.85	2.39	3.187 (7)	157
C2—H2···Cl1^iii^	0.93	2.82	3.692 (5)	156
C5—H5···O4	0.93	2.55	3.394 (7)	152
C10—H10···O6	0.93	2.46	3.318 (7)	154
C12—H12···Cl1^iv^	0.93	2.78	3.679 (5)	162
C15—H15···O4	0.93	2.58	3.185 (8)	123

Symmetry codes: (ii) *x*+1, *y*, *z*; (iii) −*x*+1, −*y*+1, −*z*+1; (iv) −*x*, −*y*+1, −*z*.
